# Tuning the activities of cuprous oxide nanostructures via the oxide-metal interaction

**DOI:** 10.1038/s41467-020-15965-8

**Published:** 2020-05-08

**Authors:** Wugen Huang, Qingfei Liu, Zhiwen Zhou, Yangsheng Li, Yunjian Ling, Yong Wang, Yunchuan Tu, Beibei Wang, Xiaohong Zhou, Dehui Deng, Bo Yang, Yong Yang, Zhi Liu, Xinhe Bao, Fan Yang

**Affiliations:** 1 State Key Laboratory of Catalysis, Dalian Institute of Chemical Physics, Chinese Academy of Sciences, 116023 Dalian, China; 2 University of Chinese Academy of Sciences, 100049 Beijing, China; 3 State Key Laboratory of Physical Chemistry of Solid Surfaces, iChEM, College of Chemistry and Chemical Engineering, Xiamen University, 361005 Xiamen, China; 4 School of Physical Science and Technology, ShanghaiTech University, 201210 Shanghai, China; 5 State Key Laboratory of Functional Materials for Informatics, Shanghai Institute of Microsystem and Information Technology, Chinese Academy of Sciences, 200050 Shanghai, China; 6 Present Address: College of Chemistry and Chemical Engineering, Chongqing University, 400044 Chongqing, China

**Keywords:** Heterogeneous catalysis, Scanning probe microscopy, Imaging techniques

## Abstract

Despite tremendous importance in catalysis, the design of oxide-metal interface has been hampered by the limited understanding of the nature of interfacial sites and the oxide-metal interaction (OMI). Through construction of well-defined Cu_2_O/Pt, Cu_2_O/Ag and Cu_2_O/Au interfaces, we find that Cu_2_O nanostructures (NSs) on Pt exhibit much lower thermal stability than on Ag and Au, although they show the same structure. The activities of these interfaces are compared for CO oxidation and follow the order of Cu_2_O/Pt > Cu_2_O/Au > Cu_2_O/Ag. OMI is found to determine the activity and stability of supported Cu_2_O NSs, which could be described by the formation energy of interfacial oxygen vacancy. Further, electronic interaction between Cu^+^ and metal substrates is found center to OMI, where the *d* band center could be used as a key descriptor. Our study provides insight for OMI and for the development of Cu-based catalysts for low temperature oxidation reactions.

## Abstract (web summary)

The design of oxide-metal interface for heterogeneous catalysis has been hampered by the limited fundamental understanding. Here, the authors demonstrate that the activities of cuprous oxide nanostructures for CO oxidation can be tuned via the oxide-metal (Cu2O/M, M = Pt, Ag, Au) interaction.

## Introduction

Metal alloy catalysts^1^ are widely used for technical applications in chemical industry^2,3^, environmental remediation^4^, and energy conversion^5^. These alloy catalysts usually consist of a precious metal component and a cheap metal component, the latter of which would segregate to the surface under oxidative conditions to form a self-limited oxide layer^6^. Such phenomena have been utilized recently for the controlled synthesis of two-dimensional (2D) oxide layer^7^. In catalysis, studies on the formation of interfacial oxide layer on precious metal surfaces could be traced back to the discovery of strong metal support interaction (SMSI)^8^ and nowadays increasingly found for metal alloy catalysts in catalytic oxidation reactions center for industrial catalysis. Despite tremendous interest, atomic understanding on the catalytic nature of these oxide layers remains a long-standing challenge because interfacial sites are minority surface sites and difficult to access. Nevertheless, the unique catalytic properties of the oxide–metal interface^9–13^ have now been recognized, although how oxide–metal interaction (OMI) tunes the properties of interfacial sites remains largely unknown.

The interface between cuprous oxide and metal has been a system of particular catalytic interest, but difficult to study owing to the dynamic nature of copper centers^14^, i.e., their valence states and coordination numbers vary with the reaction environment. Coordinatively unsaturated (CUS) metal cations have often been found as active sites for catalytic reactions. For instance, CUS Cu^+^ centers are active sites for the oxidation of ethylbenzene to acetophenone^15^. Owing to the stability and activity requirements of technological applications, stabilizing CUS centers on precious metal surfaces has been developed as an effective strategy, as demonstrated by interface-confined ferrous centers for the preferential oxidation reaction^12^. Indeed, the alloy between Cu and group VIII metals^16–20^, especially Pt, are excellent catalysts for oxygen reduction reaction, whereas alloy catalysts consisting of copper and group IB metals^21–23^ exhibit remarkable performances for the selective oxidation of hydrocarbons. Meanwhile, the formation of a copper oxide–metal interface has been generally observed on PtCu^24^, AuCu^25^, and AgCu^26^ during oxidation reactions even under mild conditions. Undoubtedly, an atomic-scale understanding on the catalytic properties of the Cu_2_O-metal (Cu_2_O-M) interface is essential to the design and improvement of Cu-based alloy catalysts for oxidation reactions.

Here, we synthesize well-defined Cu_2_O NSs on Pt(111), Au(111), and Ag(111), and study their interfacial structures and chemistry using the combination of low-temperature scanning tunneling microscopy (LT-STM), near-ambient-pressure STM (NAP-STM), X-ray photoelectron spectroscopy (XPS), and density functional theory (DFT) calculations. The atomic structures of interfacial sites are identified unambiguously with element-specific STM (ES-STM) images. Despite the same interfacial site structure, the activities of the Cu_2_O/Pt, Cu_2_O/Au, and Cu_2_O/Ag interfaces for CO oxidation are found strongly dependent on OMI, where Cu_2_O/Pt and Cu_2_O/Au are found to exhibit a reversible structural dynamics during the redox reaction at low temperatures. Our study unravels key factor in determining OMI, and subsequently the activity and stability of supported Cu_2_O NSs, which furthers our understanding on OMI and provides insight for the development of metal alloy catalysts.

## Results

### Atomic structures of Cu_2_O NSs on metal substrates

**Fig. 1:**
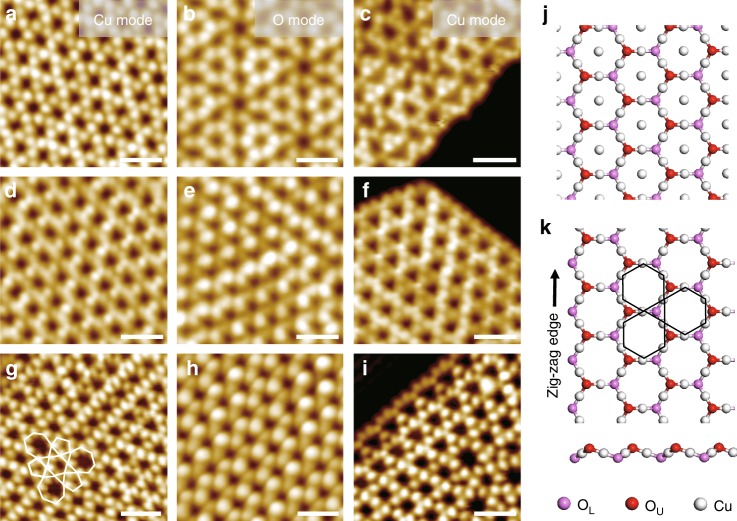
Element-specific STM images of Cu_2_O NSs grown on metal substrates. **a**–**i** STM images of **a**–**c** Cu_2_O/Pt(111), **d**–**f** Cu_2_O/Au(111), and **g**–**i** Cu_2_O/Ag(111). The surface structures of Cu_2_O NSs are displayed in the left column (Cu-mode: Cu atoms were resolved as bright dots) and the middle column (O mode: O atoms were resolved as bright protrusions) of STM images. The edge structures of Cu_2_O NSs are displayed in the right column (Cu-mode) of STM images showing zig-zag step structure with oxygen termination. 5–7 defects in Cu_2_O NS on Ag(111) are marked by white lines in **g**. **j** The structural model of Cu_2_O(111) surface. **k** The structural model of supported Cu_2_O NSs in **a**–**i**. O_U_ and O_L_ represent the upper O and lower O, respectively. All Cu_2_O NSs exhibit the hexagonal lattice of Cu_2_O(111) without dangling Cu atoms at the center of honeycomb and with an oxygen-terminated zig-zag edge. Scanning parameters: **a** *V*_s_ = −0.4 V, *I* = 1.1 nA; **b** *V*_s_ = −0.2 V, *I* = 1.4 nA; **c** *V*_s_ = −0.05 V, *I* = 3.0 nA; **d** *V*_s_ = −0.2 V, *I* = 1.5 nA; **e** *V*_s_ = −1.15 V, *I* = 1.2 nA; **f** *V*_s_ = −0.1 V, *I* = 2.1 nA; **g** *V*_s_ = −0.32 V, *I* = 0.79 nA; **h** *V*_s_ = 0.5 V, *I* = 0.1 nA; **i** *V*_s_ = −0.32 V, *I* = 0.79 nA. Scale bars: 1 nm for all images.

Cu_2_O NSs were synthesized on Pt(111), Au(111), and Ag(111) using the method described in the experimental section and display various sizes with monolayer (ML) thickness (Supplementary Fig. 1). Their atomic structures, both on the surface and at the step edge, could be clearly identified by ES-STM images^27–29^ (Fig. 1). In the Cu-mode STM image, Cu atoms were resolved as bright dots and exhibit a kagome (trihexagonal stacking) lattice. In the O-mode STM image, O atoms were imaged as protrusions and display a hexagonal lattice. Combining the Cu-mode and O-mode STM images, these Cu_2_O NSs are found to display a Cu_2_O(111)-like structure, which exhibits the honeycomb lattice of Cu_2_O(111) (Fig. 1j), but does not have dangling Cu atoms at the center of honeycomb, owing to their instability on metal substrates (Fig. 1k). The honeycomb lattice of these Cu_2_O NSs on Pt(111), Au(111), or Ag(111) displays the unit cell length (~ 6.0 Å) same as that of Cu_2_O(111). But, due to the missing of dangling Cu atoms, supported Cu_2_O NSs exhibit the stoichiometry of Cu_3_O_2_, which has been generally observed for ML Cu_2_O NSs grown on metal substrates^27,30^.

In the surface lattice of Cu_2_O NSs, 5–7 topological defects^30^ could also be observed, especially for Cu_2_O NSs supported on Ag(111) (Fig. 1g). These 5–7 topological defects were formed via the Stone-Wales type transformation of the Cu_3_O_2_ honeycomb lattice, which has been discussed previously^30^. After the annealing in ultrahigh vacuum (UHV), the step edges of Cu_2_O NSs on Pt(111), Au(111), and Ag(111) also exhibited dominantly the same O-terminated zig-zag structure, as identified by ES-STM images (Fig. 1 and Supplementary Fig. 2). On Au(111), we have shown that the O-terminated zig-zag step is thermodynamically favored^27^ and would become the dominant step structure after the annealing at 600 K, which also appears to be the case for Pt(111) and Ag(111).

Although Cu_2_O NSs on Pt(111), Au(111), and Ag(111) show the same surface and step structures, their stability was found to be influenced by OMI^31^. We found that Cu_2_O NSs on Pt(111) would start to decompose at ~470 K and decompose completely at ~630 K (Supplementary Fig. 3a, b), which was accompanied by the diffusion of Cu atoms into the bulk. Since the desorption of atomic oxygen atoms on Pt(111) would start at above 500 K^32^, the low stability of Cu_2_O NSs on Pt(111) seems to suggest that these Cu_2_O NSs could be highly active for CO oxidation. In contrast, Cu_2_O NSs appeared stable on Au(111) and Ag(111) after the annealing at 600 K (Supplementary Fig. 3c, e) although they would start to coalesce and form larger islands after the annealing at 700 K (Supplementary Fig. 3d, f). Thus, the difference in thermal stability of supported Cu_2_O NSs indicates that interfacial interaction could play a key role in determining their stability and reactivity.

### CO adsorption and reaction at the Cu_2_O–metal interface

**Fig. 2:**
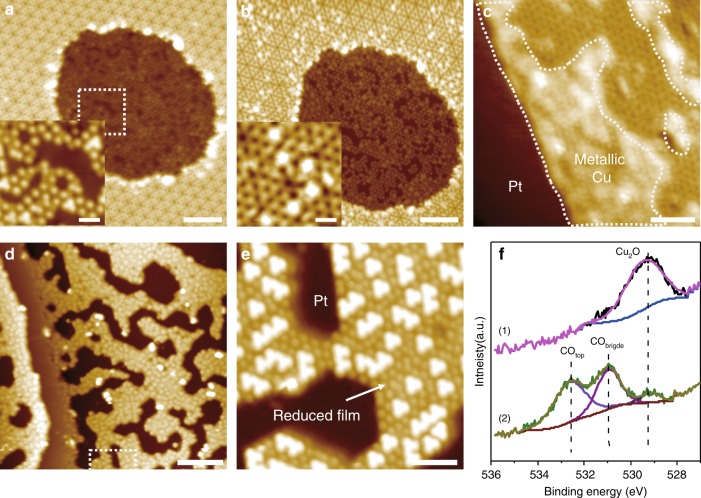
The adsorption and reaction of CO on Cu_2_O/Pt(111). **a**, **b** In situ STM images of the Cu_2_O/Pt(111) surface during the exposure to CO at 78 K. The squared region in **a** is magnified in inset, showing CO adsorption on a Pt(111) surface covered by (2 × 2)-O. Inset in **b** displays the adsorption sites of CO on Cu_2_O, which are centered at the holes of the Cu_2_O rings. **c** STM image of the partially reduced Cu_2_O/Pt(111) surface after the surface in **b** was annealed to 300 K. The reduction by CO started from both the step edge and the surface of Cu_2_O. The dotted curves indicate the regions of metallic Cu. **d**, **e** STM images of a Cu_2_O/Pt(111) surface after the exposure to 1 × 10^−7^ mbar CO for 5 min at 300 K. High-resolution STM image (**e**) from the marked area in **d** shows the reduction of Cu_2_O by CO led to the formation of triangular Cu_3_O_x_ clusters, sitting on a metallic layer. **f** XPS O 1*s* spectra of the Cu_2_O/Pt(111) surface before (1) and after (2) the exposure to 1.2 × 10^−7^ mbar CO for 5 min at 300 K. Lattice O of Cu_2_O with binding energy at 529.4 eV has been mostly consumed after CO exposure, accompanying the adsorption of CO on top sites (CO_top_) and bridge sites (CO_bridge_) of the exposed Pt surface. Scanning parameters: **b** *V*_s_ = −0.3 V, *I* = 0.2 nA; inset: *V*_s_ = −0.3 V, *I* = 0.5 nA; **c** *V*_s_ = −0.15 V, *I* = 1.2 nA; **e** *V*_s_ = −0.15 V, *I* = 0.5 nA. Scale bars: **a**, **b** 5 nm, inset 1 nm; **c** 3 nm; **d** 10 nm; **e** 2 nm.

The adsorption and reaction of CO were then compared for Cu_2_O NSs on Pt(111), Au(111), and Ag(111). When the Cu_2_O/Pt(111) surface was exposed to CO at 78 K, CO adsorbed randomly on Pt(111) and the surface of Cu_2_O NSs remained clean, indicating a weak interaction between CO and Cu_2_O (Fig. 2a and Supplementary Fig. 4). Continuous CO exposure at 78 K could cause the appearance of CO molecules on the surface domains of Cu_2_O (Fig. 2b). But, analysis of high-resolution STM image showed that these CO molecules were located dominantly on Pt sites exposed at the center of the hexagonal Cu_2_O rings (Supplementary Fig. 5), owing to the much stronger chemisorption of CO on Pt^33^. The large holes of the Cu_2_O rings allow for CO adsorption on Pt sites even when Pt(111) is fully covered by Cu_2_O.

The CO-covered surface of Cu_2_O/Pt(111) was then annealed to 300 K in UHV. Figure 2c showed the reduction of Cu_2_O NSs by CO, starting from both the surface terrace of Cu_2_O and the step edges of Cu_2_O. From the development of reaction front, the reaction rate of Cu_2_O reduction from the step edges of Cu_2_O was found to be faster (~1.3 times) than that on the surface terrace of Cu_2_O (Supplementary Fig. 6). The faster reduction rate along the steps of Cu_2_O could be attributed to the lower coordination numbers of O atoms at step edges. Nonetheless, CO oxidation at both the step edge and the surface terrace of Cu_2_O occurred via an interfacial dual-site reaction mechanism, i.e., CO adsorbed on Pt sites to react with neighboring lattice oxygen in Cu_2_O.

Consistently, when the Cu_2_O/Pt(111) surface was exposed to CO at 300 K, the reduction of Cu_2_O could start right upon the exposure to 5 × 10^−8^ mbar CO and the exposure to 1 × 10^−7^ mbar CO for 5 min at 300 K could lead to the complete decomposition of Cu_2_O layer into metallic Cu atoms or triangular Cu_3_O_x_ clusters (Fig. 2d–e). These Cu_3_O_x_ clusters sit on a metallic layer with its apparent height (~2 Å, Supplementary Fig. 7) approximating the step height of Cu(111). The metallic layer exhibited a hexagonal lattice with a non-uniform lattice spacing (~5 Å), which is approximately twice that of Cu(111) and indicating the formation of a PtCu_3_ alloy (Supplementary Fig. 7d). The exothermic reaction of CO oxidation could facilitate the alloying between Cu and Pt since the formation of PtCu_3_ is a thermodynamically favored process^34^. Accordingly, XPS O 1*s* spectra (Fig. 2f) showed that only ~10% of Cu_2_O was left when 0.6 ML Cu_2_O/Pt(111) was exposed to CO at 300 K. CO exposure also led to the adsorption of CO on bridge and top sites of Pt(111), as shown in the O 1*s* spectra^35^. In comparison, when the Pt(111) surface is fully covered by Cu_2_O, the reduction of the Cu_2_O layer requires a higher CO pressure at 5 × 10^−6^ mbar (Supplementary Fig. 8). The major problem that prevents Pt group metals from catalyzing low temperature CO oxidation is the strong chemisorption (poisoning) of CO blocks surface sites for the adsorption and activation of O_2_. The superior activity of supported Cu_2_O NSs demonstrated above showed that such problem could be annihilated by the formation of a Cu_2_O/Pt interface.

**Fig. 3:**
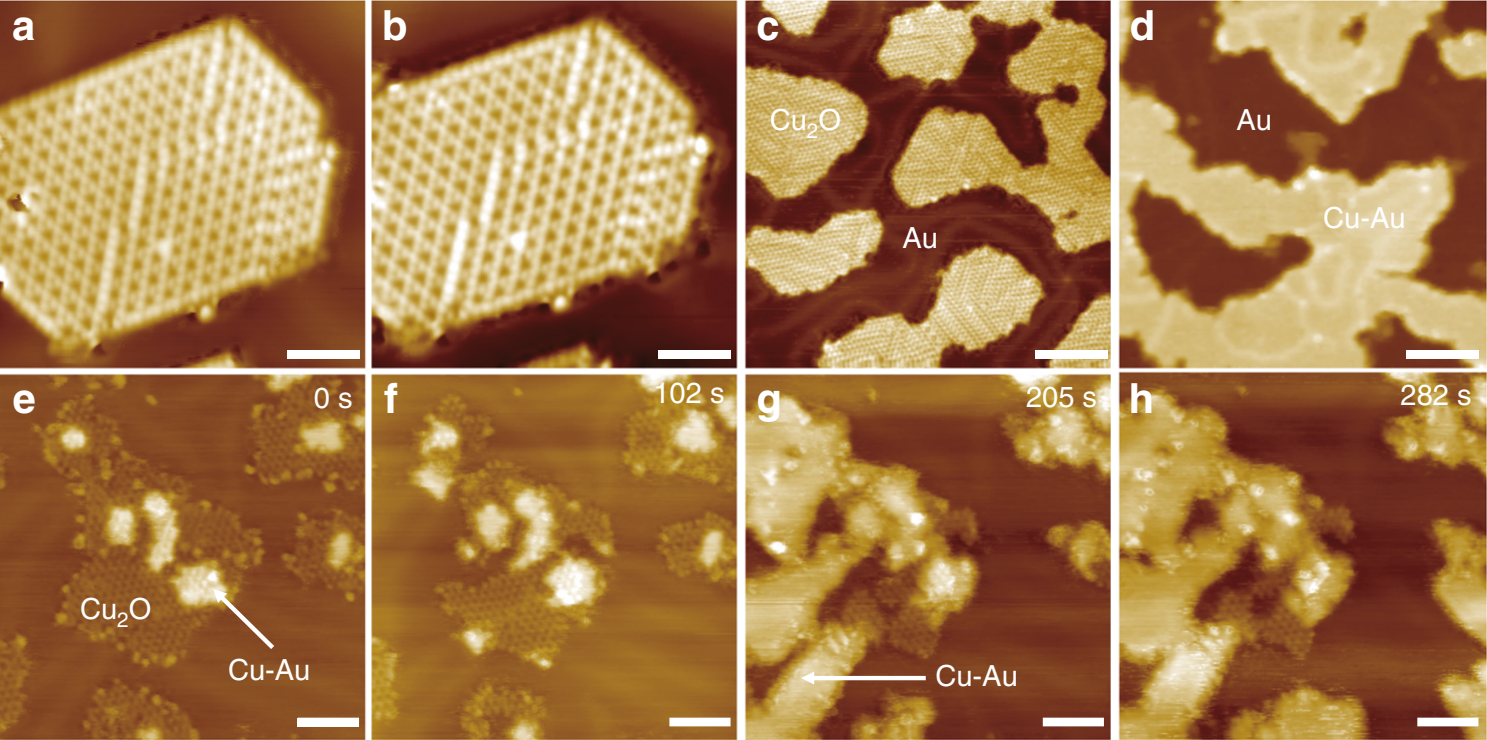
The adsorption and reaction of CO on Cu_2_O/Au(111). **a**, **b** In situ STM images before (**a**) and after (**b**) the exposure of CO on Cu_2_O/Au(111) at 78 K. No adsorption was obvious after the exposure of 18 L CO. **c**, **d** Ex-situ and **e**–**h** in situ NAP-STM images on the reduction of Cu_2_O on Au(111) in 1 mbar (**d**) or 0.5 mbar (**e**–**h**) CO at 300 K. The reduction of Cu_2_O NSs led to the formation of a Cu–Au surface alloy. The reaction times were marked at the top right of images. Scanning parameters: **a** *V*_s_ = −2.0 V, *I* = 0.06 nA; **b** *V*_s_ = −1.7 V, *I* = 0.03 nA; **c** *V*_s_ = 1.1 V, *I* = 0.09 nA; **e** *V*_s_ = 1.2 V, *I* = 0.02 nA; **f** *V*_s_ = 1.2 V, *I* = 0.05 nA. Scale bars: **a**, **b** 2 nm; **c**, **d** 10 nm; **e**–**h** 5 nm.

In comparison, when the Cu_2_O/Au(111) surface was exposed to CO at 78 K (Fig. 3a, b), no adsorption of CO molecules was obvious in STM and Cu_2_O NSs remained intact. While CO molecules could adsorb on bulk Cu_2_O surfaces at cryogenic temperatures^36^, the interaction between CO and supported Cu_2_O NSs appeared to be much weakened by OMI. Owing to the weak interaction between CO and Cu_2_O/Au(111), CO exposure under vacuum conditions did not cause any change on the Cu_2_O/Au(111) surface at 300 K. However, as the partial pressure of CO was raised to 0.5 mbar, enhanced CO adsorption could cause the continuous reduction of Cu_2_O NSs to form the Cu–Au surface alloy (Fig. 3c–h). In situ NAP-STM images showed further that the reduction started from the edges of Cu_2_O NSs on Au(111) (Supplementary Fig. 9). When the Au(111) surface was fully covered by Cu_2_O, the onset pressure for the reduction of the Cu_2_O layer needs be further raised to ~6 mbar CO in our NAP-STM study. Thus, both the activity and the stability of supported Cu_2_O NSs are influenced by OMI.

**Fig. 4:**
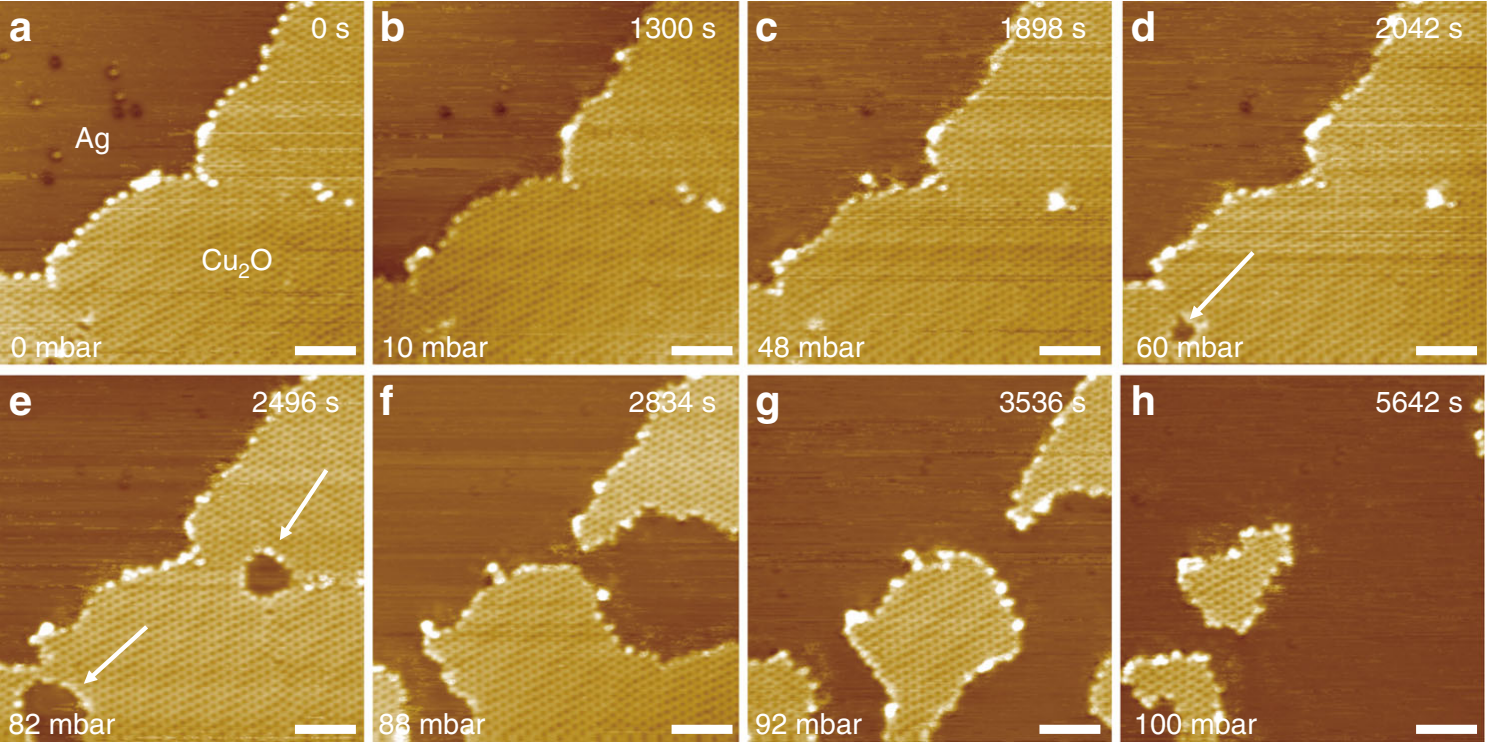
Reaction of CO on Cu_2_O/Ag(111). **a**–**h** In situ NAP-STM images on the reduction of the Cu_2_O/Ag(111) surface under elevated CO pressures from 0 to 100 mbar at 300 K. Reduction of the Cu_2_O layer was observed in >48 mbar CO, starting from domain boundaries indicated by white arrows in **d**, **e**. The pressures of CO were labeled at the bottom left of STM images, and reaction times were labeled at the top right of images. Scanning parameters: **a**–**h** *V*_s_ = 0.9 V, *I* = 0.1 nA. Scale bars: 5 nm for all images.

While CO oxidation occurred readily at the Cu_2_O/Pt(111) and the Cu_2_O/Au(111) interface under UHV and NAP conditions, respectively, no appreciable activities of CO oxidation were observed on the Cu_2_O/Ag(111) surface in 10 mbar CO at 300 K (Fig. 4 and Supplementary Fig. 10), despite Cu_2_O exposes the same zig-zag edge structure on Ag(111) as those on Pt(111) and Au(111) and the adsorption energy of CO on Ag(111) is similar to that on Au(111)^37^. Indeed, even when CO pressure was raised to ~48 mbar, the zig-zag edges of Cu_2_O on Ag(111) could not react with CO at 300 K, whereas one could start to observe the reduction of Cu_2_O from low-coordination sites at the domain boundaries of Cu_2_O. The inactivity of the zig-zag edges of Cu_2_O on Ag(111) is in drastic contrast to the active zig-zag edges of Cu_2_O on Au(111) and Pt(111), where the onset reaction pressures are in the ranges of 10^−1^ and 10^−8^ mbar CO, respectively. Thus, the reactivity trend of the Cu_2_O/M interface follows the order of: Cu_2_O/Pt(111) > Cu_2_O/Au(111) > Cu_2_O/Ag(111). Note that, the rate equation could be affected by both the surface coverage of CO and the activation energy. Since the adsorption energies of CO are approximately similar (−0.29 eV)^37^ on Au(111) and Ag(111), the reactivity comparison between Cu_2_O/Au(111) and Cu_2_O/Ag(111) could indeed rule out influence of surface CO coverage and corroborated further the critical role of OMI in tuning the activation barrier and thus reactivity of the oxide–metal interface.

The regeneration of Cu_2_O NSs is important to complete the catalytic cycle of CO oxidation. We found that the re-oxidation of the Pt–Cu surface alloy on CO-covered Pt(111) requires only an O_2_ atmosphere at 5 × 10^−8^ mbar (at >400 K) to form well-ordered Cu_2_O NSs (Supplementary Fig. 11a), whereas the Au–Cu surface alloy remained metallic in 5 × 10^−7^ mbar O_2_ at 450 K (Supplementary Fig. 11b). But, Cu_2_O NSs were found to grow from the steps of the Au–Cu surface alloy on Au(111) in ~1 mbar O_2_ at 300 K (Supplementary Fig. 11c). Similarly, Cu on Ag(111) could be oxidized into Cu_2_O in 0.17 mbar O_2_, accompanying the oxidation of Ag(111) (Supplementary Fig. 11d). In this case, CO oxidation on the Cu_2_O/Ag(111) surface could be a convoluted process since Ag(111) could also be oxidized by O_2_ under NAP conditions^38^. Yet, the redox cycle of supported Cu_2_O NSs should exhibit a lower barrier on Pt(111) than on Au(111) and Ag(111). Although group IB precious metals do not suffer from the CO-poisoning problem, their weak interaction with CO and O_2_^39,40^ are not favorable for maintaining an active Cu_2_O–metal interface, which appears crucial for a sustained catalytic activity in low temperature CO oxidation. Owing to the strong chemisorption of CO on Pt group metals, the presence of excess O_2_ or the increase of reaction temperature should be beneficial to sustain the active Cu_2_O–metal interface for CO oxidation.

### Catalytic performance for CO oxidation

To examine the role of OMI in tuning the catalytic activity of supported Cu_2_O NSs, we prepared further PtCu, AuCu and AgCu alloy nanoparticle catalysts supported on carbon black (CB) and measured their catalytic performances in CO oxidation as a function of temperature and under O_2_-rich conditions (oxide supports were avoided to reduce the complexity of interfacial interaction). Previous studies^24,26,41^ have shown that Cu atoms in bimetallic nanoparticles would segregate to the surface to form oxide layer under oxidative conditions, which is typical for cheap metals in bimetallic alloys^6,12,42^. The chemical states of these catalysts were confirmed further in the post-reaction analysis using quasi-in situ XPS (Supplementary Fig. 12), where the XPS chamber is attached with a high-pressure reactor for catalytic studies. XPS measurements showed that, after reaction, Cu in the PtCu/CB and AuCu/CB catalysts remained as Cu_2_O from the Cu 2*p* and Cu Auger spectra^43^, whereas Cu in the AgCu/CB catalyst was oxidized into CuO. The size and structure of alloy nanoparticle catalysts were examined by transmission electron microscopy (TEM, Supplementary Fig. 13). The particle morphology appeared similar before and after reaction, except that the ripening of particles could be observed.

**Fig. 5:**
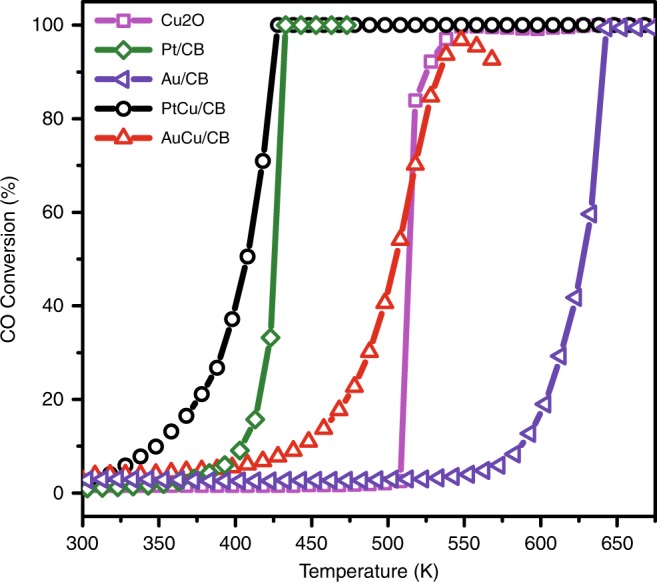
Catalytic performance of the PtCu/CB, Pt/CB, AuCu/CB, Au/CB and Cu_2_O catalysts. The light-off curves for CO oxidation were obtained under the condition of 1% CO, 20% O_2_, and 79% He. Space velocity was 60,000 mL g^−1^ h^−1^.

Figure 5 compared the light-off curves of the series of catalysts in CO oxidation, where the catalytic performances of Cu_2_O powders and Pt, Au, and Ag nanoparticles supported on CB (denoted as Pt/CB, Au/CB, and Ag/CB, respectively) were also measured for comparison. Consistent with the above model studies, the PtCu/CB catalyst exhibited appreciable activity for CO oxidation right above 300 K, whereas Pt/CB showed neglectable CO conversion at below the CO desorption temperature (~400 K)^44^. The activity of the AuCu/CB catalyst is also superior to those of Cu_2_O and Au/CB catalysts, and showed activity at above 300 K, although the increase of CO conversion is slower than that of PtCu/CB catalyst. The activities of AgCu/CB and Ag/CB catalysts (Supplementary Fig. 14) were not plotted with above catalysts for comparison, since the operation of these catalysts did not suffer CO poisoning and involved surface oxidation of Ag^38,45^ (as evidenced in Supplementary Fig. 11). As Ag alone showed good activity for CO oxidation under O_2_-rich conditions, the formation of CuO_x_/Ag interface reduced the activity of Ag for CO oxidation, which could be attributed to the covering of active Ag sites and consistent with the above STM study. Weak OMI in Cu_2_O/Ag is insufficient to maintain the active Cu^+^ state, leading to the formation of CuO (Supplementary Fig. 12) and the decrease of reactivity. By comparison, the formation of Cu_2_O/M interfaces indeed annihilate the CO poisoning problem suffered by Pt group metals and the reactivity from powder catalytic measurements agree with the model studies in the order of: Cu_2_O/Pt > Cu_2_O/Au > Cu_2_O/Ag, which again confirmed the activities of Cu_2_O NSs are tuned by OMI.

### Analysis on the oxide–metal interaction

**Fig. 6:**
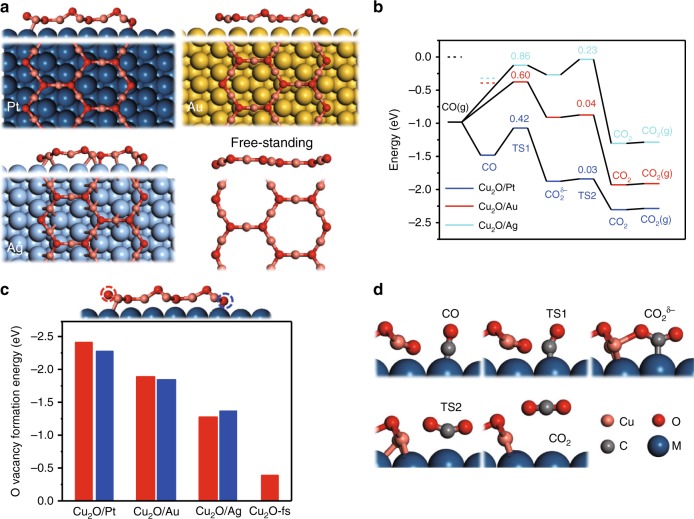
DFT calculations about CO oxidation reaction on supported Cu_2_O NSs. **a** DFT-optimized structures of Pt(111), Au(111), Ag(111) supported and freestanding Cu_8_O_6_ nanoribbons (denoted as Cu_2_O/M (M=Pt, Au, Ag) and Cu_2_O-fs). **b** Potential energy diagram for the reaction between CO and O on the stabilized edge of M(111) supported Cu_2_O NSs. The value of barrier is labeled above the corresponding transition state (unit: eV). The energy of CO(g) is represented as the Gibbs free energy of CO under UHV conditions (1 × 10^−7^ mbar) while the black dashed bar at 0 eV marks the DFT-calculated energy of CO without corrections. The red and cyan dashed bars mark the adsorption energy of CO on the top site of Au(111) and Ag(111) surface near the edge of Cu_2_O NSs. **c** The formation energy of O vacancy for two kinds of O atoms, marked by red and blue dashed circles, at the edges of Cu_2_O/M and Cu_2_O-fs. **d** The optimized structures for reaction intermediates and transition states (adsorbed CO, transition state 1 (TS1), CO_2_ adsorbed with bending configuration, transition state 2 (TS2) and CO_2_ adsorbed with straight configuration) in **b**.

Spin-polarized DFT calculations were further conducted to understand the nature of OMI in tuning the activities of supported Cu_2_O NSs. Based on the atomic structures determined from ES-STM, structural models of M(111)-supported (M = Pt, Au, Ag) and freestanding Cu_8_O_6_ nanoribbons were constructed, denoted as Cu_2_O/M and Cu_2_O-fs. After structural optimization, these models showed the honeycomb lattice of an O–Cu–O tri-layer with O-terminated zig-zag edges (Fig. 6a), consistent with STM results. To understand the above model catalytic studies, we looked into the reaction between CO and O of supported Cu_8_O_6_ nanoribbons (Fig. 6b–d). STM study (Supplementary Fig. 6) showed that the reduction of Cu_3_O_2_ occurred mainly at edge O sites. We thus considered mainly the reaction between CO and edge O atoms in supported Cu_8_O_6_ nanoribbons. The energy of CO in gas phase was corrected as the Gibbs free energy of CO under UHV conditions (1 × 10^−7^ mbar).

For the Cu_2_O/Au and Cu_2_O/Ag systems, the Gibbs free energy barrier for transition state 1 (TS1, Fig. 6b, d) corresponds to the energy difference between TS1 and CO in gas phase since CO adsorption is not preferred under UHV conditions and the free energy barrier increases linearly to the decrease of the logarithm of CO pressure (Supplementary Figs. 15, 16). The reaction diagrams (Fig. 6b, d) suggested that the rate determining step is the formation of CO_2_, where the barrier on Cu_2_O/Pt (0.42 eV) is lower than those on Cu_2_O/Au (0.60 eV) and Cu_2_O/Ag (0.86 eV). The high activity of the Cu_2_O/Pt interface could be interpreted via a dual-site mechanism^12,46^ that Pt(111) provides proper CO adsorption while the interface between Pt and edge Cu sites enables O_2_ dissociation (Supplementary Fig. 17) via a low dissociation barrier (0.39 eV), which is also thermodynamically favored. In comparison, O_2_ dissociation at Cu_2_O/Au and Cu_2_O/Ag interfaces are thermodynamically less-favorable, although the process was still enhanced by OMI, when compared with those on Au(111) and Ag(111)^37^.

We then look further into geometric and electronic factors that could be utilized to quantify the role of OMI in dictating the activity of Cu_2_O/M systems. Typically, the adhesion energy (*E*_adh_) has been used to quantify OMI, which for Cu_8_O_6_ nanoribbon on M(111), gives the order of Cu_2_O/Pt (−0.76 eV) > Cu_2_O/Ag (−0.71 eV) > Cu_2_O/Au (−0.58 eV). This is obviously inconsistent with the reactivity order observed in the above experimental studies and indicates that the dual interaction of Cu/O atoms with M(111) could be not directly relevant to the key reaction steps, i.e., the formation of CO_2_ and the subsequent formation of O vacancy in Cu_2_O. In this case, oxygen vacancy formation energy (*E*_Ovf_) should be of direct relevance and indeed we found the order of *E*_Ovf_ for supported Cu_2_O NSs tracks the same order as their catalytic activities (Cu_2_O/Pt > Cu_2_O/Au > Cu_2_O/Ag, Fig. 6c). Further, compared with *E*_Ovf_ on the edge of Cu_2_O-fs (−0.39 eV), *E*_Ovf_ for supported Cu_2_O NSs are much more negative, in the range of −1.33 ~ −2.35 eV, suggesting the formation of oxygen vacancy is much facilitated at the edges of Cu_2_O/M. Thus, OMI plays a key role in weakening the Cu-O bond and facilitating the removal of O atoms by CO. The stronger OMI, the easier the removal of O and the formation of CO_2_.

**Fig. 7:**
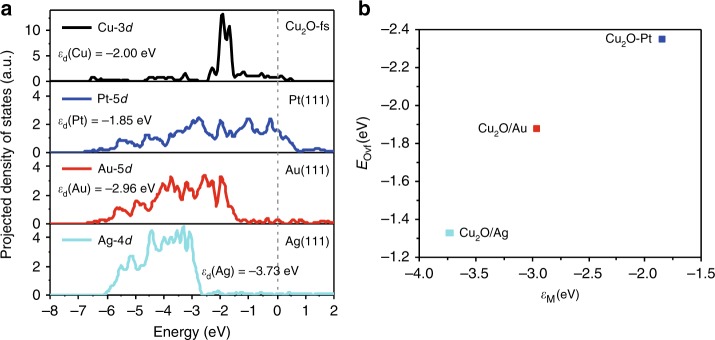
Electronic interaction between Cu_2_O-fs and M(111). **a** Density of states projected on *d* orbitals of Cu atoms on the edge of Cu_2_O-fs and M atoms from the surface layer of M(111) (M = Pt, Au, Ag) and their corresponding *d* band centers. The grey dashed line marks the Fermi level. **b** *E*_Ovf_ for O atoms at the edge of Cu_2_O/M as a function of *d* band center of M(111).

As we looked into the process of O removal, the interaction between M(111) and Cu^+^ (the formation of Cu-M bond after the removal of O, Supplementary Fig. 18) is found as a key descriptor for the formation of O vacancies (Fig. 7). We calculated the projected density of states on *d* orbitals of Cu atoms on the edge of Cu_2_O-fs and M atoms from the surface layer of M(111) clean surface (M = Pt, Au, Ag) and their corresponding *d* band centers (*ε*_d_, Fig. 7a). According to the *d*-band theory^47^, when the *ε*_d_ of M is closer to the Fermi level, the stronger interaction of metal surface with Cu^+^ could be expected since the *d* band for Cu^+^ is relatively localized (molecule like, Fig. 7a). Therefore, the bonding strength between M(111) and Cu^+^ follows the order of Cu_2_O/Pt > Cu_2_O/Au > Cu_2_O/Ag, consistent with the rank order of *E*_Ovf_ and the catalytic activity. Quantitatively, we use the distance between *ε*_d_ of Cu_2_O and *ε*_d_ of M(111) to describe the electronic interaction between Cu^+^ and M. Assuming *ε*_d_ of Cu_2_O at a fixed value, the differences in electronic interaction among Cu_2_O/M could be represented by the differences in *ε*_d_ of M. As we plotted *E*_Ovf_ as a function of *ε*_d_ for M(111) in Fig. 7b, an excellent scaling relationship could be observed. Note that, geometric factors, such as surface rumpling (Δ*h*), has also been used to describe the formation energy of O vacancies on oxide surfaces.^48^ But, our study found that surface rumpling cannot be used to describe the Cu_2_O–M interface, because the order of Δ*h* for Cu_8_O_6_ nanoribbons on metal substrates is: Cu_2_O/Pt (0.46 Å) > Cu_2_O/Ag (0.40 Å) > Cu_2_O/Au (0.34 Å), inconsistent with their reactivity order. Therefore, OMI should be described by the electronic interaction between Cu_2_O and metal substrates, where *ε*_d_ could be used as a simple descriptor. Pt group metals are favored over group IB metals for interfacial catalysis of CO oxidation, due to the much stronger OMI that lowers/stabilizes greatly the reaction energy surfaces. We identify the interaction between M(111) and Cu^+^ as a key descriptor to quantify the role of OMI in dictating the reactivity of Cu_2_O/M in CO oxidation. The stronger interaction of M(111) with Cu^+^ enables the higher activity of Cu_2_O/M for catalytic oxidation.

To understand the catalytic properties of the Cu_2_O–metal interface, well-defined Cu_2_O NSs were synthesized on a series of M(111) (M = Pt, Au, Ag) single crystals and studied at the atomic scale using LT-STM, NAP-STM, XPS, and DFT calculations. The surface and step structures of supported Cu_2_O NSs were identified unambiguously in ES-STM images, which show the same Cu_3_O_2_ honeycomb lattice for the above Cu_2_O NSs, with similar unit cell length and a dominant O-terminated zig-zag edge. Despite their structural similarity, supported Cu_2_O NSs exhibit a substrate-dependent thermal stability. Cu_2_O NSs on Pt(111) showed a surprisingly low stability, with an onset decomposition temperature even lower than the desorption temperature of oxygen atoms on Pt(111). In contrast, Cu_2_O NSs on Ag(111) and Au(111) remained stable in UHV even after the annealing at up to 700 K.

As we compared the reactivities of Cu_2_O NSs on M(111) for CO oxidation, we found all surfaces could react with CO at room temperature. But, the reduction of Cu_2_O on Pt(111) needs only a CO pressure at 5 × 10^−8^ mbar, whereas the reduction of Cu_2_O on Au(111) requires a CO pressure at 0.5 mbar, and even much higher on Ag(111). Vice versa, the regeneration of Cu_2_O NSs needs only 5 × 10^−8^ mbar O_2_ on Pt(111), while the regeneration of Cu_2_O NSs requires NAP conditions on Au(111) (~1 mbar O_2_) and Ag(111) (~0.2 mbar O_2_). Further catalytic tests on powder samples corroborated that the interface with Cu_2_O NSs could annihilate the CO-poisoning problem and enable low temperature CO oxidation on Pt group metals. The reactivity of supported Cu_2_O NSs is found determined and much enhanced by OMI, when compared with Cu_2_O-fs. The analysis of reaction mechanism and OMI showed that OMI facilitates CO oxidation by enhancing the activity of interfacial oxygen atoms and stabilizing oxygen vacancies in Cu_2_O NSs. The stronger the OMI, the lower the barrier for CO oxidation catalyzed by supported Cu_2_O NSs. The role of OMI in dictating the activity and stability of supported Cu_2_O NSs could be described by the formation energy of interfacial oxygen vacancy. Further, *d* band center could serve as a simple descriptor for OMI to correlate the electronic interaction between Cu^+^ and metal substrates with the formation energy of oxygen vacancy and the reactivity of Cu_2_O/M interfaces for CO oxidation. Thus, our study provides insight for OMI and a strategy for the design of oxide NS catalysts for oxidation reactions.

## Methods

### Model catalyst synthesis and characterization

The experiments were carried out in two UHV systems. The first system consists of a CreaTec LT-STM with base pressure at <4 × 10^−11^ mbar and a preparation chamber equipped with XPS, and cleaning facilities. LT-STM measurements were conducted at 78 K, using an electrochemically etched W tip. The second system consists of a SPECS NAP-STM with base pressure <2 × 10^−10^ mbar and a preparation chamber equipped with cleaning facilities. NAP-STM measurements were conducted at 300 K using a Pt-Ir tip. Pt(111), Au(111), and Ag(111) single crystals (Mateck) were cleaned by cycles of Ar^+^ sputtering at 1.2 kV and annealing to 800 K for Au(111) and Ag(111) or 1000 K for Pt(111) until no impurities were obvious on the surface from STM.

Well-ordered Cu_2_O NSs of ML thickness could be synthesized on metal single crystals by evaporating Cu in 1 × 10^−7^ mbar O_2_ at cryogenic temperatures, which were followed by the annealing in O_2_ at ~500 K. To obtain well-ordered structure, Cu_2_O/Au(111) and Cu_2_O/Ag(111) could be annealed in UHV condition above 500 K. On Au(111), Cu_2_O NSs could be prepared by depositing Cu in ~3 × 10^−7^ mbar NO_2_ at 300 K, which were followed by the annealing in NO_2_ at 400 K. Cu_2_O/Ag(111) could also be prepared by oxidation of Cu/Ag(111) by near ambient pressure of O_2_ at 300 K. Then to remove the surface Ag–O species, Cu_2_O/Ag(111) was annealed at ~450 K. For all experiments, O_2_ and CO were purified by liquid nitrogen for over 1 h to remove impurities before being introduced into the chambers. Different modes of ES-STM images were acquired using W tip and controlling the indentation of W tip into the Cu_2_O surface to obtain a CuO_x_-terminated tip^29,49^. All STM images were processed with SPIP software (Image Metrology). XPS characterization of Cu_2_O/Pt(111) was conducted with Mg-kα energy source (*hv* = 1253.6 eV). The binding energies were calibrated with that of Pt 4*f*_7/2_ signal located at 71.0 eV.

Powder catalysts preparation and catalytic tests. PtCu/CB catalyst was prepared by co-impregnation method. First, 100 mg CB (Vulcan XC-72) were dispersed into 5 mL H_2_O by ultrasonic treatment for 0.5 h. Next the aqueous solutions of H_2_PtCl_6_ and Cu(NO_3_)_2_ were successively added. The nominal loading of Pt was 4 wt%, and the molar ratio of Cu to Pt was 1:4. Then, excess water was evaporated at room temperature under continuous agitation (500 rpm). Finally, the solid was dried at 333 K in an oven for 12 h to obtain the catalyst for use. The preparation procedures for AuCu/CB and AgCu/CB catalysts were exactly the same as that for PtCu/CB by only replacing the precursor into HAuCl_4_ or AgNO_3_ respectively. Pt/CB, Au/CB, and Ag/CB were prepared in similar manner but without addition of Cu(NO_3_)_2_. The Cu_2_O powder (99% purity) was bought from Energy Chemical.

CO oxidation was carried out in a fixed-bed reactor. The reactant gas consisted of 1% CO, 20% O_2_, and 79% He (Messer). The reactant gas was flowed at 20 mL min^−1^ through 20 mg catalyst to achieve a space velocity of 60,000 mL g^−1^ h^−1^. The tail gas was analyzed online by gas chromatography equipped with methane converter in front of flame ionization detector. Before reactions, all catalysts were reduced in high-purity H_2_ flow at 473 K for 2 h. After cooling down in high-purity Ar flow, samples were kept in reactant gas for 1 h at 298 K, then temperature-dependent CO oxidation was performed from room temperature to 573–673 K at a rate of 1 K min^−1^.

XPS analysis of powder catalysts were performed in ThermoFischer ESCALAB 250Xi photoelectron spectrometer using monochromated X-ray irradiation Al-Kα (*hv* = 1486.7 eV) and 180° double focusing hemispherical analyzer with a six-channel detector. The binding energies of the photoemission spectra were calibrated to C 1*s* peak at 284.4 eV. The XPS chamber is attached with a high-pressure reactor for catalytic studies. Samples were reduced in pure hydrogen (1 bar) to 473 K for 1 h and then cooled to 300 K in H_2_. CO oxidation reaction was then performed in the mixture gas (1 bar, 1% CO and 20% O_2_ with He balance) to 400 K for PtCu/CB or 500 K for AuCu/CB and AgCu/CB. After reaction, the sample was cooled to room temperature in reactant gas. XPS measurements were performed after evacuating the reactor cell and transferring the samples in UHV into the analyzer chamber without air exposure.

### Computational method

Theoretical calculations were carried out based on density functional theory (DFT) framework by using Vienna ab initio simulation packages (VASP)^50^ with the projector augmented wave (PAW)^51^ scheme and the plane-wave basis set with the cutoff energy of 400 eV were adopted^52^. The generalized gradient approximation (GGA) functional developed by Perdew−Burke−Ernzerhof (PBE)^53^ for the exchange-correlation term was used. The method of Methfessel-Paxton was employed to define the partial occupancies of metal-supported or freestanding Cu_8_O_6_ (each supercell includes 8 Cu and 6 O) ribbon. Climbing image nudged elastic band (NEB) method^54,55^ was used for searching of transition states. All the models were well converged until the forces decreased to less than 0.05 eV Å^−1^. Models of Cu_8_O_6_ nanoribbons on Pt(111), Au(111), and Ag(111) were adopted. For each model, a 4-layer 2 × 4√3 supercell of M(111) (M = Pt, Au, Ag) surface was used as substrate. Gamma-centered K-mesh of 4 × 1 × 1 was employed for structure optimization or transition-state searching. 4-layer 4 × 4 M(111) (M = Pt, Au, Ag) surface with Gamma-centered K-mesh of 4 × 4 × 1 were adopted as reference of pure metal for CO adsorption calculations. For all models, the vacuum was set to be about 12 Å. During structure optimization, the bottom layer of substrate was constrained while the rest parts were fully relaxed. Freestanding Cu_8_O_6_ nanoribbon was fully relaxed at the fixed lattice in bulk value (6.105 Å) with the Gamma-centered K-mesh 4 × 1 × 1.

The *d* band center (*ε*_d_) was calculated through the formula^56^ below:

1 $$\varepsilon _{\mathrm{d}} = \frac{{\int \nolimits_{ - \infty }^{\infty} n_{\mathrm{d}}\left( \varepsilon \right)\varepsilon {\mathrm{d}}\varepsilon }}{{\int \nolimits_{ - \infty }^{\infty} n_{\mathrm{d}}\left( \varepsilon \right){\mathrm{d}}\varepsilon }},$$

where *ε* is energy and $n_{\mathrm{d}}\left( \varepsilon \right)$ is the density of states of electrons. Integral interval is from negative infinity to positive infinity.

Surface rumpling was defined as $\Delta h$, which is calculated through the formula:

2 $$\Delta h = \frac{{\sum \nolimits_{i = 1}^n \left| {h_{{\mathrm{Oi}}} - \overline {h_{{\mathrm{Cu}}}} } \right|}}{n},$$

where $h_{{\mathrm{Oi}}}$ is the height of the ith O, ${\overline {h_{{\mathrm{Cu}}}}}$ is the average height of Cu, *n* is the number of O in Cu_8_O_6_ nanoribbon.

Adhesion energy *E*_adh_ was calculated through the formula:

3 $$E_{{\mathrm{adh}}} = E_{{\mathrm{tot}}} - E_{{\mathrm{oxi}}} - E_{\mathrm{M}},$$

where *E*_tot_ is DFT-calculated total energy for M(111) supported Cu_8_O_6_ nanoribbon; *E*_oxi_ and *E*_M_ are energy for freestanding Cu_8_O_6_ nanoribbon and M(111), respectively.

Oxygen vacancy formation energy (*E*_Ovf_) is the energy needed to remove one oxygen from Cu_8_O_6_ nanoribbons, referencing to CO and CO_2_ in gas phase, which is calculated through the formula below:

4 $$E_{{\mathrm{Ovf}}} = E_{{\mathrm{Ovac}}} + E_{{\mathrm{CO}}_2} - E_{{\mathrm{CO}}} - E_{{\mathrm{per}}},$$

where $E_{{\mathrm{Ovac}}}$ and $E_{{\mathrm{per}}}$ are DFT-calculated total energy for structures with one oxygen vacancy and without that, respectively; $E_{{\mathrm{CO}}_2}$ and $E_{{\mathrm{CO}}}$ are energy for CO_2_ and CO in gas phase.

The Gibbs free energy of CO ($G_{{\mathrm{CO}}}$) in gas phase is calculated through the formula below:

5 $$G_{{\mathrm{CO}}} = E_{{\mathrm{elec}}} + {\mathrm{ZPE}} + \int_{}^{}C_{p}{\mathrm{d}}T - {\mathrm{TS}} + kT{\mathrm{lnp}}_{CO}/p_0,$$

where $E_{elec}$ is the electronic energy calculated via VASP; ZPE is the zero-point energy, $\int C_{p}{\mathrm{d}}T$ is the variety of isobaric heat capacity as the temperature changes, *S* is entropy and *T* is 298 K. $p_{{\mathrm{CO}}}$ and $p_0$ is the pressure of CO and the standard atmosphere (1000 mbar) respectively; *k* is the Boltzmann constant. The results of ZPE, $\int_{}^{}C_{p}{\mathrm{d}}T$, *S* is given by the ASE (atomic simulation environment) code^57^.

## Supplementary information

Supplementary Information

## Data Availability

All data supporting this work are available from the corresponding author upon reasonable request.
