# Supplementary material for: Tuning the activities of cuprous oxide nanostructures via the oxide-metal interaction
Source: Nat Commun. 2020 May 8;11:2312. doi: 10.1038/s41467-020-15965-8 (PMC7210313; doi:10.1038/s41467-020-15965-8)
Supplement: Supplementary file 1 — Supplementary Information [file 41467_2020_15965_MOESM1_ESM.pdf]

# Supplementary Information

## Tuning the Activities of Cuprous Oxide Nanostructures via the Oxide-Metal Interaction

Huang et al.

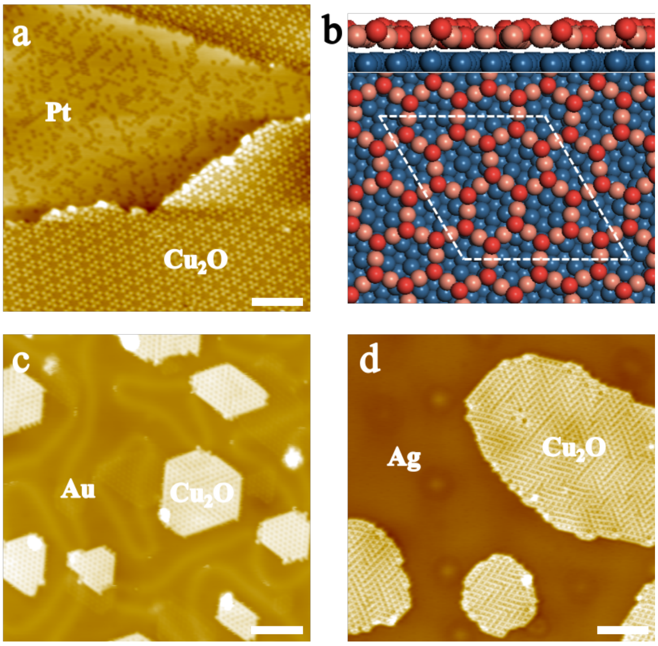


**Supplementary Figure 1.** Large-Scale STM images of $Cu_2O$ NSs on metal substrates. (a) $Cu_2O$/Pt(111), (c) $Cu_2O$/Au(111) and (d) $Cu_2O$/Ag(111). (a) To obtain the well-ordered $Cu_2O$ structure, as-deposited cuprous oxide was annealed at above 450 K. $Cu_2O$ NSs on Pt(111) was annealed in $O_2$ to prevent the decomposition of $Cu_2O$. (b) Structural model of $Cu_2O$ film on Pt(111) with a ($\sqrt{43}\times\sqrt{43}$)R7.6-$Cu_3O_2$ supercell. (c-d) $Cu_2O$ were epitaxially grown on Au(111) and Ag(111). Red, coral pink and blue balls are O, Cu and Pt atoms, respectively. Scanning parameters: (c) $V_s = -2.0$ V, $I = 0.1$ nA; (d) $V_s = -1.0$ V, $I = 0.1$ nA. Scale bars in (a), (c) and (d) are 5 nm.

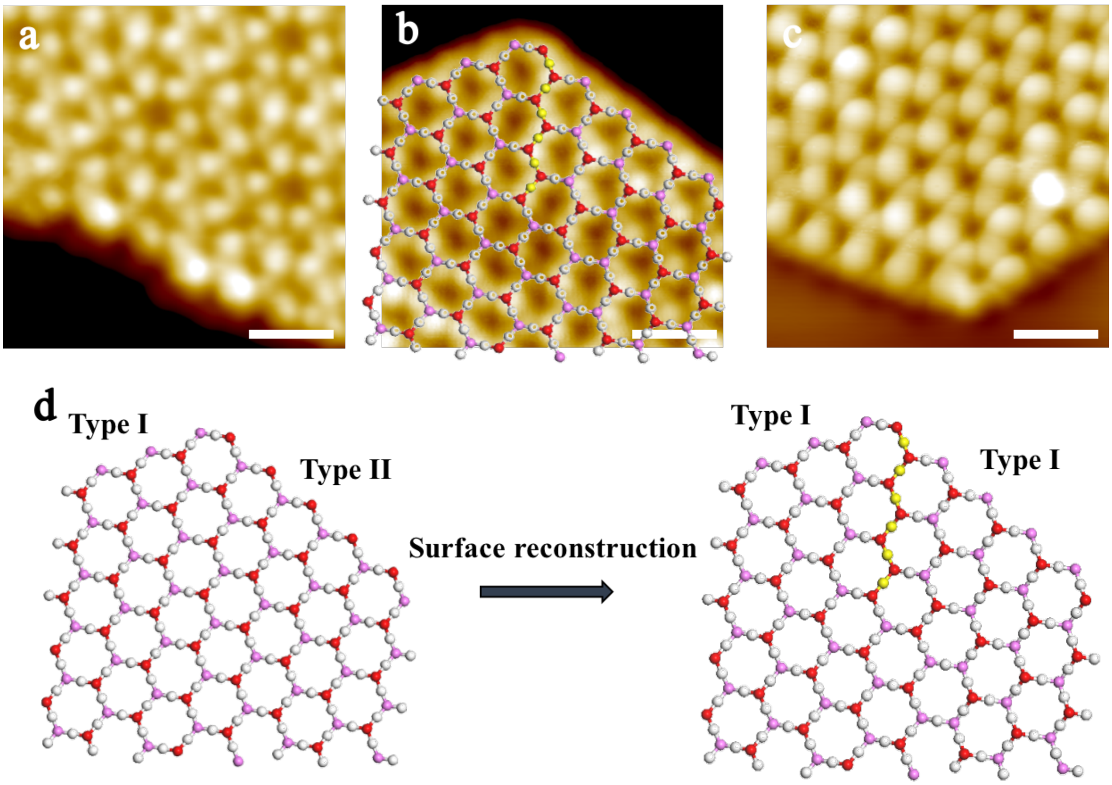


**Supplementary Figure 2.** O-mode STM images on the edge structures of $Cu_2O$ NSs on metal substrates. (a) $Cu_2O$/Pt(111), (b) $Cu_2O$/Au(111) and (c) $Cu_2O$/Ag(111). It is clear that all edges have an O-terminated zig-zag edge structure. (d) After surface reconstruction, $O_u$ terminated edges (type II) were transformed into more stable $O_L$ terminated edges (type I), as illustrated in (b). Scanning parameters: (a) $V_s = -0.05$ V, $I = 0.48$ nA; (b) $V_s = -1.15$ V, $I = 1.2$ nA; (c) $V_s = 0.5$ V, $I = 0.1$ nA. Scale bars: 1 nm for all images.

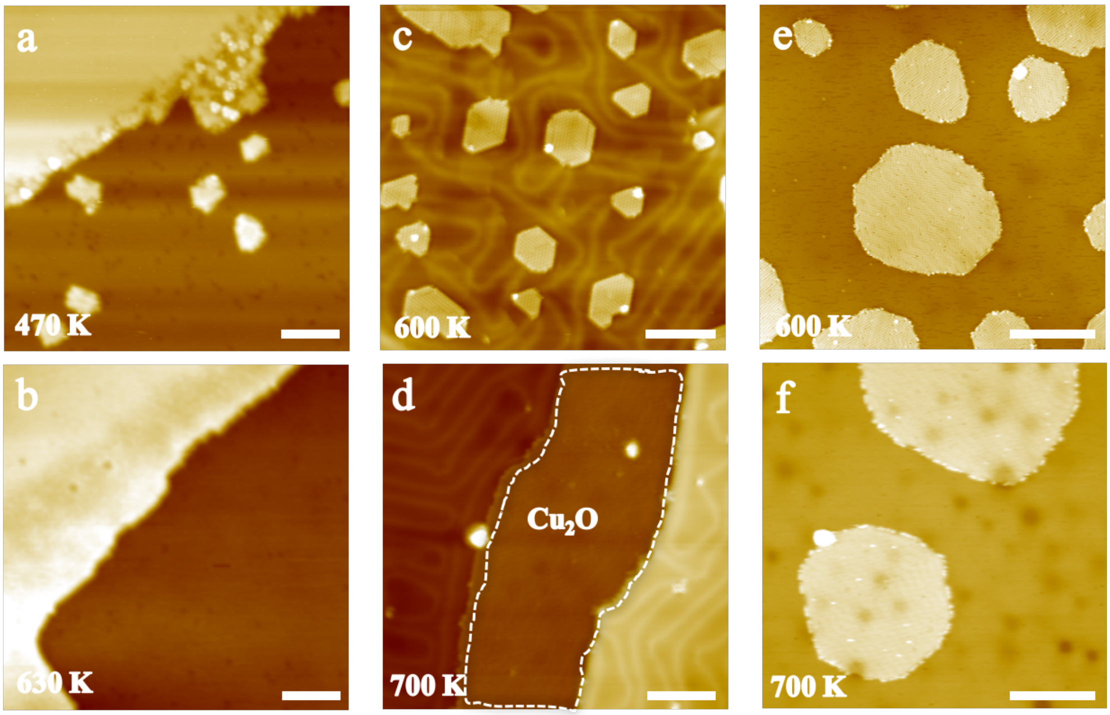


**Supplementary Figure 3.** The thermal stability of $Cu_2O$ NSs on metal substrates. STM images of (a-b) $Cu_2O$/Pt(111), (c-d) $Cu_2O$/Au(111) and (e-f) $Cu_2O$/Ag(111) after the annealing in UHV at different temperatures. The annealing temperatures were labeled in the bottom left corner of the STM image. On Pt(111), $Cu_2O$ NSs started to decompose at ~ 470 K and a clean metallic surface was obtained after the annealing at ~ 630 K. In contrast, $Cu_2O$ NSs on Au(111) and Ag(111) remained stable at 600 K and started to coalesce at 700 K. Dashed lines in (d) indicated the region of $Cu_2O$ layer on Au(111). Scale bars: (a-b) 5 nm; (c-d) 10 nm; (e-f) 20 nm.

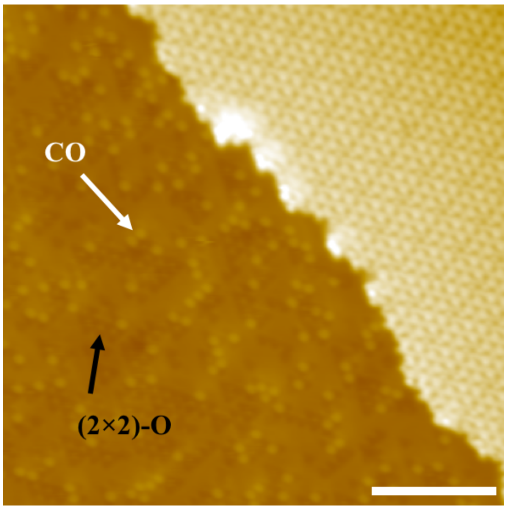


**Supplementary Figure 4.** STM image on the adsorption of CO on $Cu_2O$/Pt(111). CO molecules adsorbed randomly on the exposed surface of Pt(111). The surface was partially covered with (2×2)-O adatoms (marked by the arrow) formed during the deposition of $Cu_2O$ on Pt(111). There was no adsorption of CO molecules on the surface or at the edge of the $Cu_2O$ layer. Scanning parameters: $V_s = -0.2$ V, $I = 1.0$ nA. Scale bar is 5 nm.

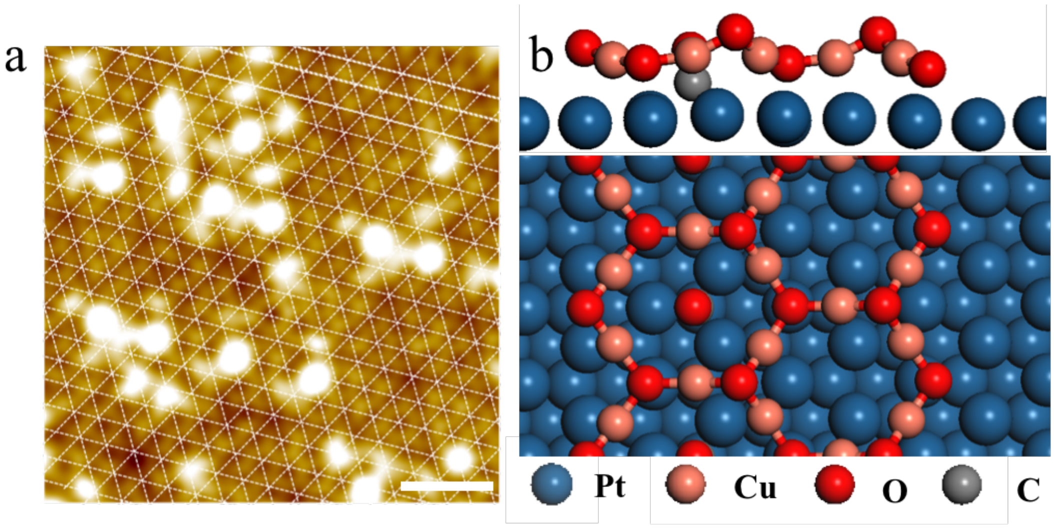


**Supplementary Figure 5.** CO adsorption on the surface of $Cu_2O$/Pt(111) at 78 K. (a) STM image with a super-imposed hexagonal grid to show the adsorption sites of CO. Each cross marks the center of a $Cu_2O$ ring. CO molecules were found to adsorb on Pt atoms exposed at the center of the hexagonal $Cu_2O$ ring. Scanning parameters: $V_s = 0.3$ V, $I = 0.13$ nA. Scale bar is 2 nm. (b) The corresponding structural model for CO adsorption on $Cu_2O$/Pt(111). The calculated adsorbed energy of CO in the center of the rings is -1.38 eV.

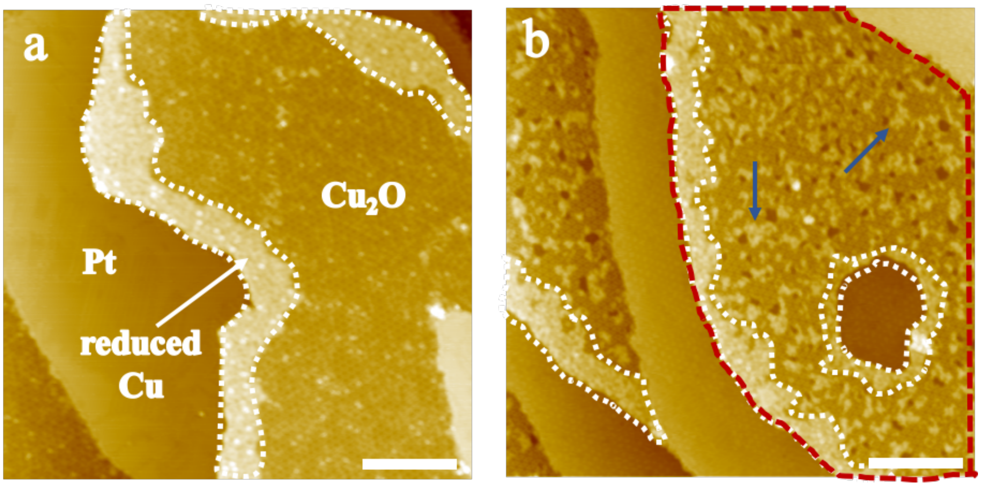


**Supplementary Figure 6.** Comparison of the reduction of $Cu_2O$/Pt(111) after CO exposure at 78K, followed by the annealing to 300 K. The amount of CO exposure is (a) 0.35 L and (b) 5.6 L. The areas marked by white dashed lines were metallic Cu domains formed upon CO reduction at the $Cu_2O$/Pt interface. CO reduction also took place from inside the surface plane of $Cu_2O$, forming Cu islands as indicated by blue arrows. In the region marked by red dashed lines in (b), the ratio of Cu areas at the $Cu_2O$/Pt interface (areas marked by white dashed lines) to that in the surface plane (as indicated by blue arrows) was ~ 1.3, suggesting a faster reduction rate at the $Cu_2O$/Pt interface. Scale bars: (a-b) 20 nm.

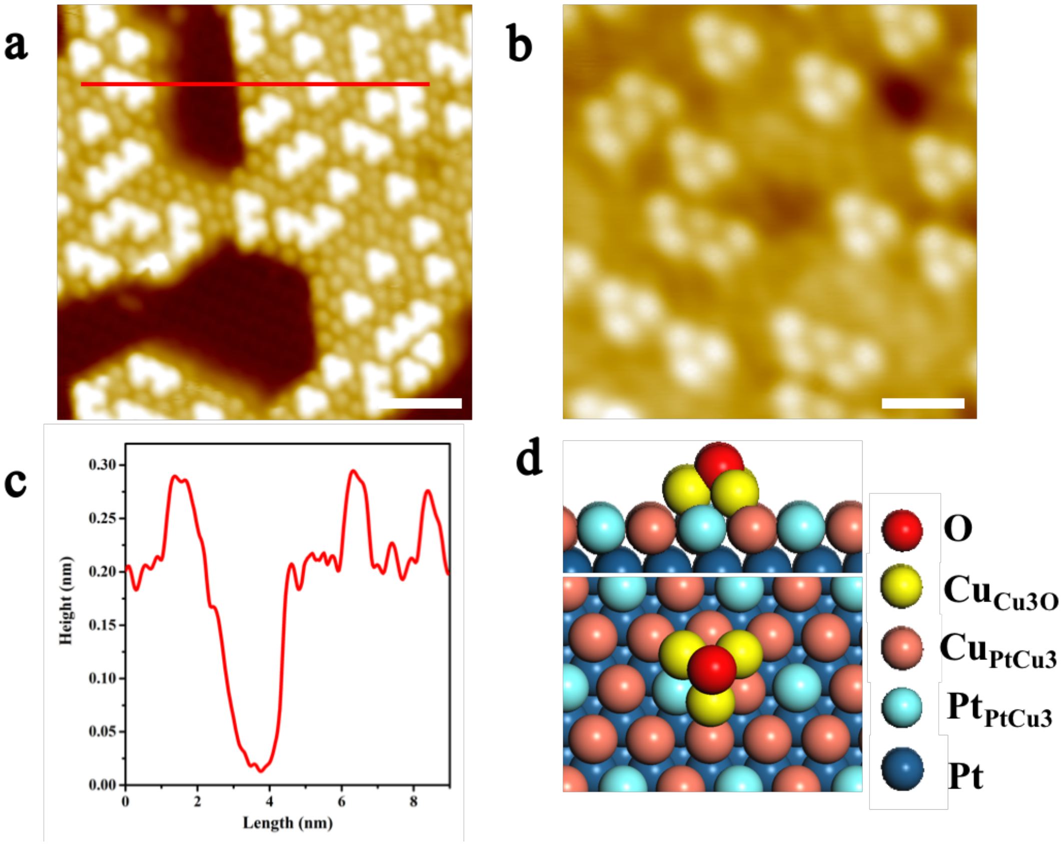


**Supplementary Figure 7.** STM images and the corresponding structural model of the reduced $Cu_2O$/Pt(111) surface. (a-b) STM images of $Cu_2O$/Pt(111) after the reaction with CO at 300 K acquired with different tip conditions. (c) The corresponding height profile along the line drawn in (a) indicates the $Cu_2O$ layer was reduced to a metallic film, with an apparent height of ~ 2 Å. The structural model was proposed based on the atomic distances measured from the supported triangular clusters and the metallic film underneath (with a lattice spacing of ~ 5 Å) and illustrated in (d). The $Cu_2O$ layer was reduced by CO at 300 K into $Cu_3O_x$ clusters sitting on a $PtCu_3$(111) film. Scanning parameters: (a) $V_s = -0.15$ V, $I = 0.5$ nA; (b) $V_s = -0.4$ V, $I = 1.1$ nA. Scale bars: (a) 2 nm; (b) 1 nm.

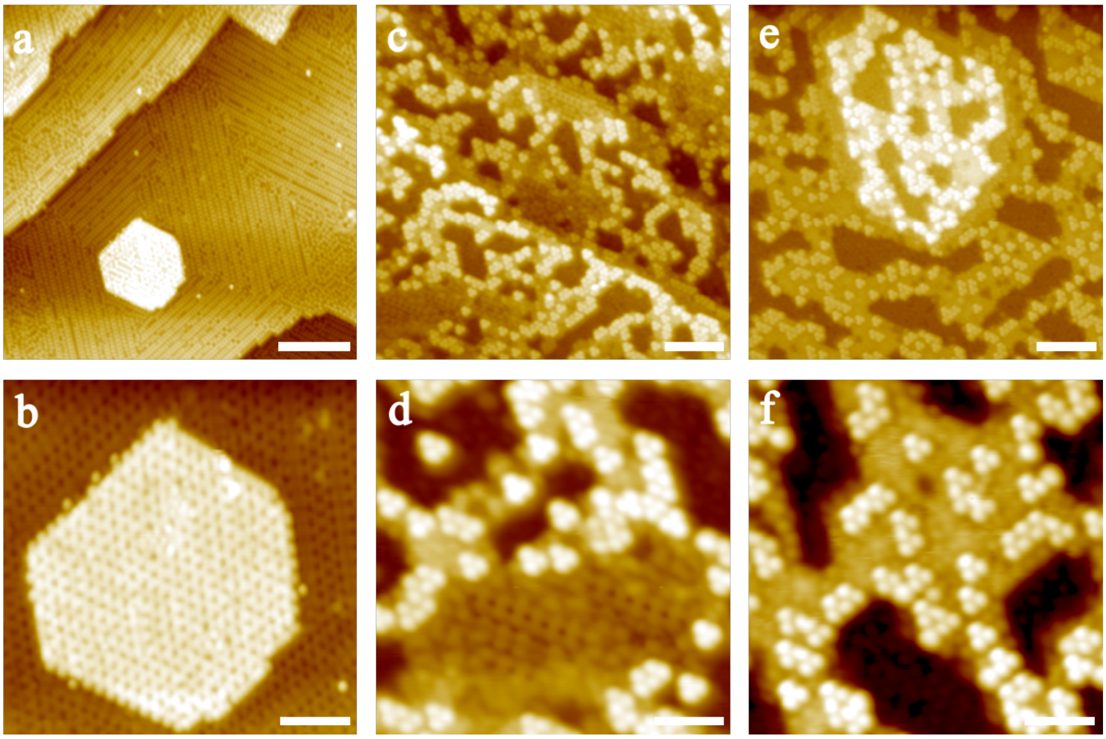


**Supplementary Figure 8.** Reaction of CO on ~ 1.5 ML $Cu_2O$ layer on Pt(111). STM images of $Cu_2O$/Pt(111) (a-b) before and (c-f) after the exposure to CO at 300 K. The $Cu_2O$ Film was reduced partially by $5 \times 10^{-7}$ mbar CO in (c-d) and reduced completely by $5 \times 10^{-6}$ mbar CO in (e-f). Scanning parameters: (b) $V_s = -0.05$ V, $I = 1.0$ nA; (d) $V_s = -1.0$ V, $I = 0.1$ nA; (f) $V_s = -1.0$ V, $I = 0.5$ nA. Scale bars: (a) 10 nm; (b) 3 nm; (c, e) 5 nm; (d, f) 2 nm.

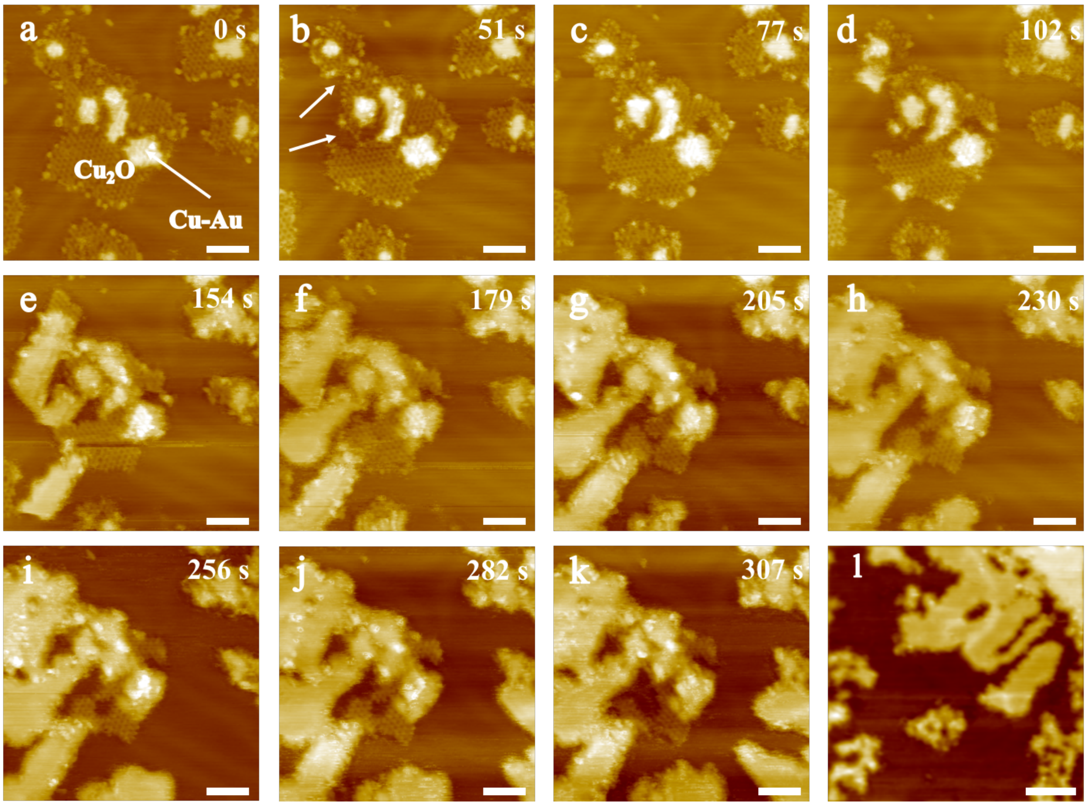


**Supplementary Figure 9.** Reaction of CO on $Cu_2O$/Au(111). (a-k) Consecutive in-situ STM images of the $Cu_2O$/Au(111) surface during the exposure of 0.5 mbar CO at 300 K. (l) Large-scale STM image after CO reduction at 300 K. The reaction times were marked in the top right of images. Arrows in (b) indicated the reduction front. Scale bars: (a-k) 5 nm; (l) 10 nm.

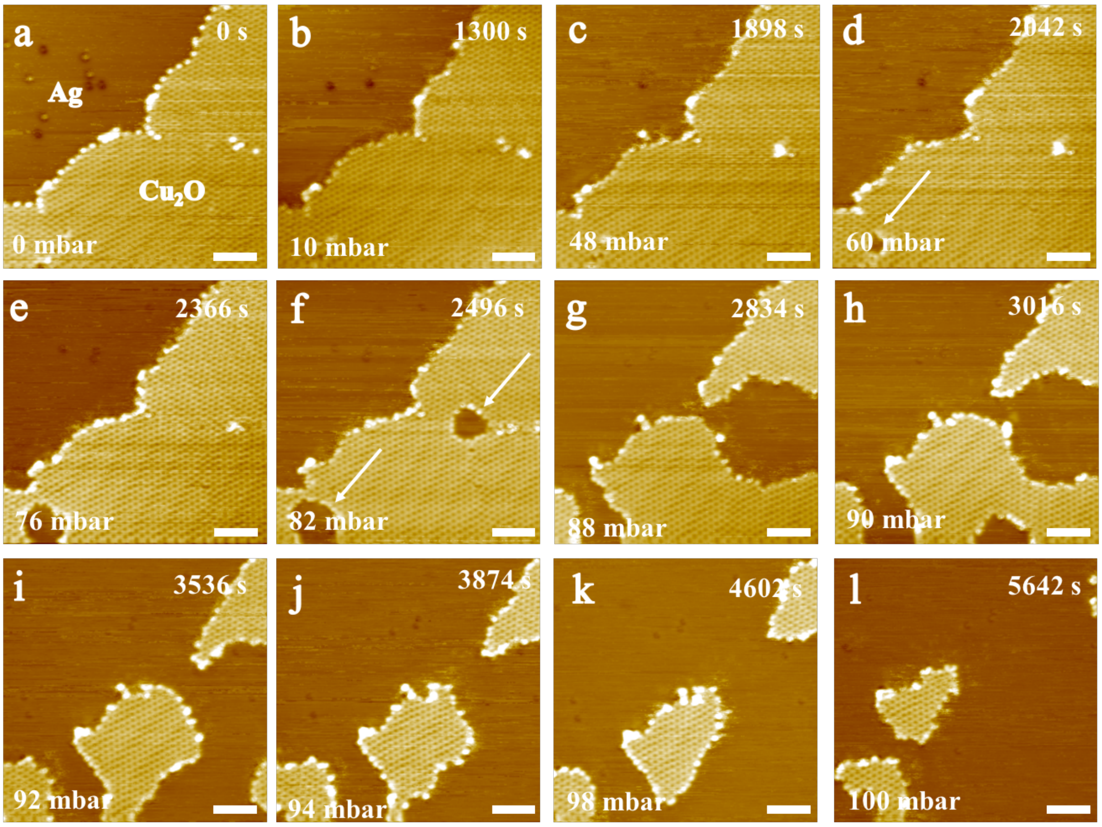


**Supplementary Figure 10.** Reaction of CO on $Cu_2O$/Ag(111). (a-l) Consecutive in-situ AP-STM images during the reduction of $Cu_2O$/Ag(111) under elevated CO pressures from 0-100 mbar at 300 K. Scanning parameters: $V_s = 0.9$ V, $I = 0.1$ nA. Scale bars: (a-l) 5 nm.

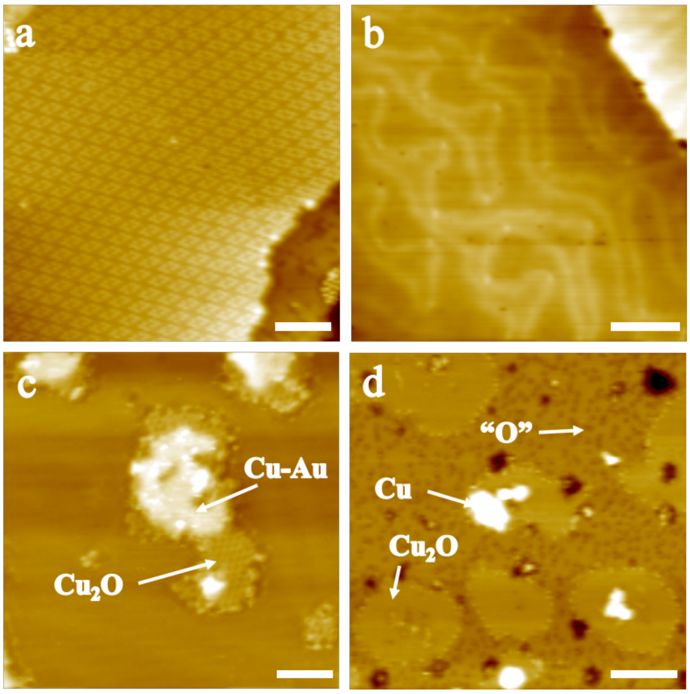


**Supplementary Figure 11.** STM images on the regeneration of $Cu_2O$ NSs on metal substrates. (a) Well-ordered $Cu_2O$ NSs were recovered on Pt(111) by annealing the PtCu alloy surface on Pt(111) in $5 \times 10^{-8}$ mbar $O_2$ at 480 K. (b) The AuCu alloy surface on Au(111) remained metallic after the annealing in $5 \times 10^{-7}$ mbar $O_2$ at 450 K. (c) The AuCu alloy surface on Au(111) was re-oxidized in 1 mbar $O_2$ at 300 K to form ordered $Cu_2O$ NSs developed from step edges of metal alloy islands. (d) Oxidation of Cu islands on Ag(111) in 0.17 mbar $O_2$ at 300 K leading to the formation of $Cu_2O$ NSs on Ag(111) and oxide-like structures of Ag (marked as "O"). Scale bars: (a, c) 5 nm; (b, d) 10 nm.

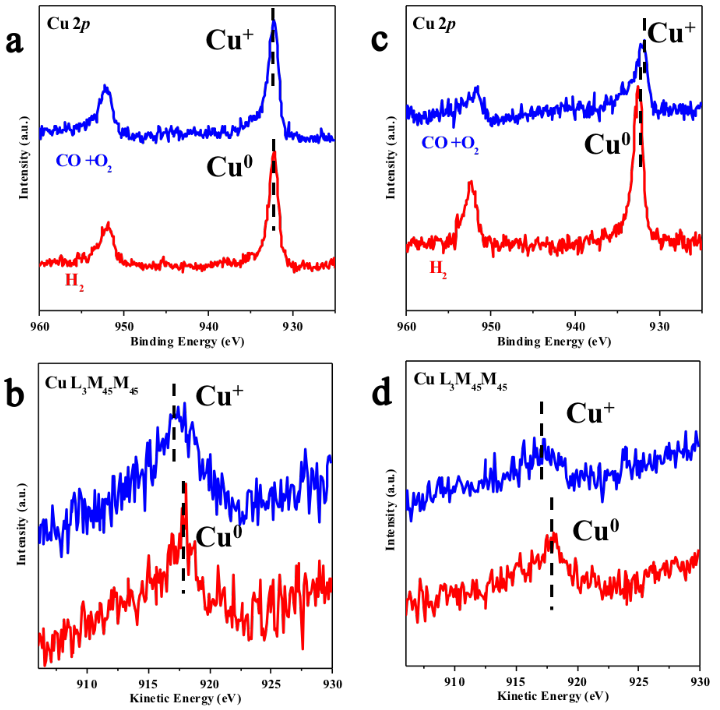


**Supplementary Figure 12.** Quasi-in situ XPS spectra of various alloy catalysts. (a) Cu 2*p* and (b) Cu LMM spectra of PtCu/CB catalyst; (c) Cu 2*p* and (d) Cu LMM spectra of AuCu/CB catalyst; (e) Cu 2*p* spectra of AgCu/CB catalyst (Cu LMM spectra were not shown due to their very weak signal). The red lines show XPS spectra measured after the catalysts were reduced by $H_2$ before catalytic reaction measurements. The blue lines show XPS spectra measured after the catalysts were tested for CO oxidation in the mixture gases of CO and $O_2$. Combining Cu 2*p* and Cu LMM spectra, XPS results suggested that $Cu_2O$ was formed as an active phase for PtCu/CB and AuCu/CB catalysts during the catalytic CO oxidation reaction. However, CuO was formed (or coexisted with $Cu_2O$) for AgCu/CB catalyst during the CO oxidation reaction.

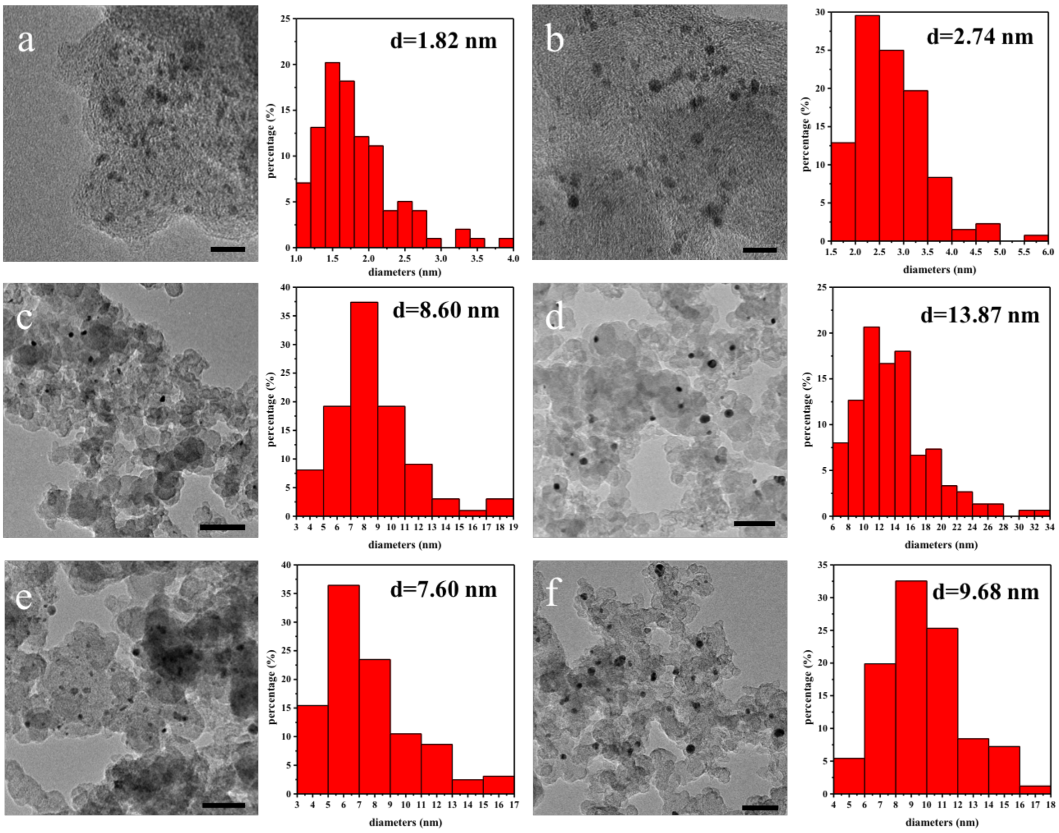


**Supplementary Figure 13.** TEM analysis of alloy catalysts. Representative TEM images and corresponding size distributions of (a-b) PtCu/CB, (c-d) AuCu/CB and (e-f) AgCu/CB catalysts measured (a, c, e) after the reduction by $H_2$ or (b, d, f) after catalytic tests of CO oxidation. After catalytic tests, sizes of nanoparticles increased. The size distribution analysis was done by counting over 100 nanoparticles. Scale bars: (a-b) 10 nm; (c-d) 100 nm; (e) 50 nm; (d) 60 nm.

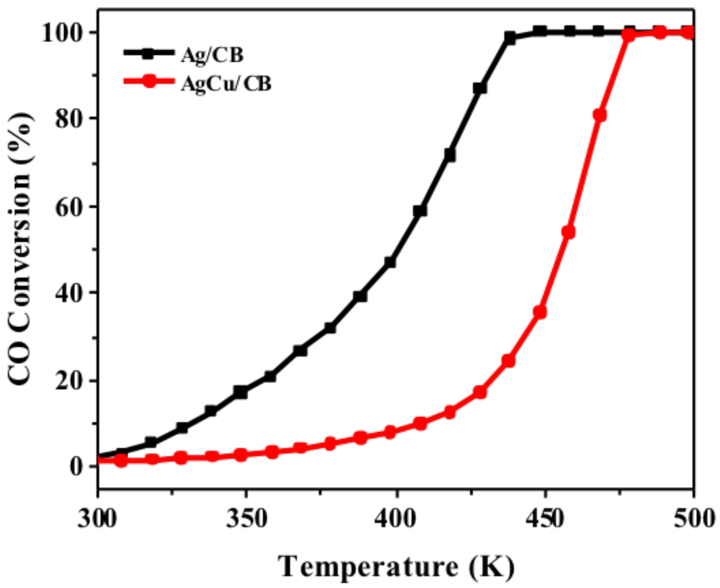


**Supplementary Figure 14.** Light-off curves of AgCu/CB and Ag/CB catalysts measured under the condition of 1% CO, 20% $O_2$, and 79% He. Space velocity was 60000 $mL\cdot g^{-1}\cdot h^{-1}$. The Ag/CB catalyst exhibited excellent activity, whereas the formation of AgCu reduced the activity of Ag catalyst.

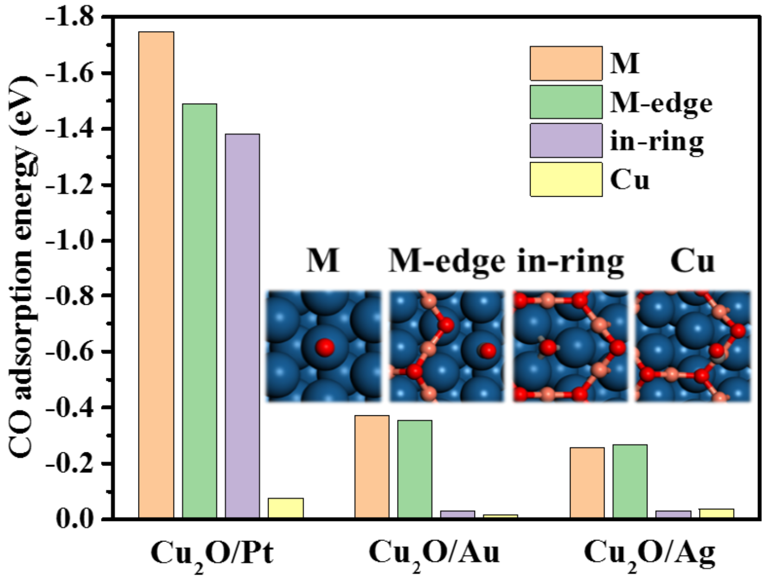


**Supplementary Figure 15.** Adsorption energies of CO on the top sites of clean M(111) (M=Pt, Au, Ag), near edge sites of $Cu_2O$ NSs on M(111), in the oxide ring on M(111), and on Cu sites of $Cu_2O$ NSs.

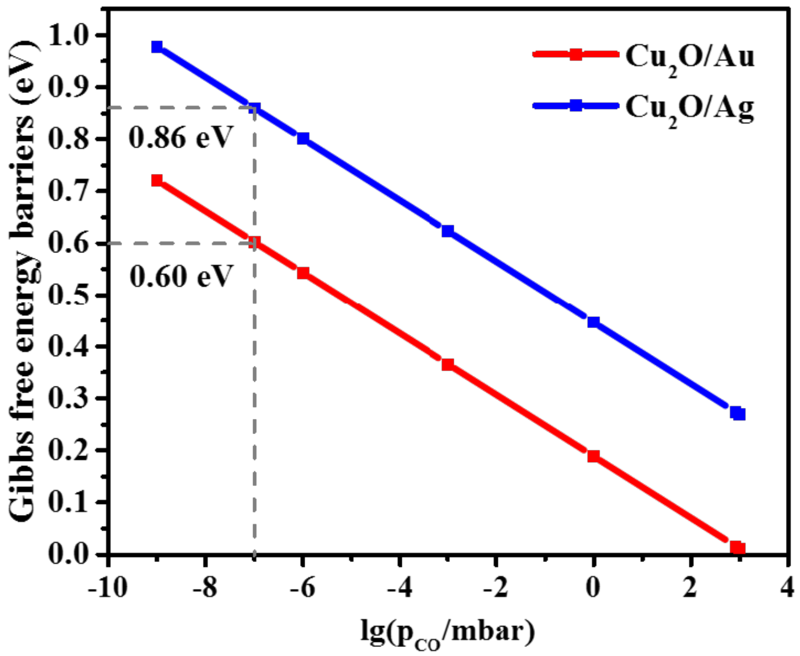


**Supplementary Figure 16.** Gibbs free energy barriers for CO oxidation on $Cu_2O$/Au and $Cu_2O$/Ag as a function of the logarithm of CO pressure.

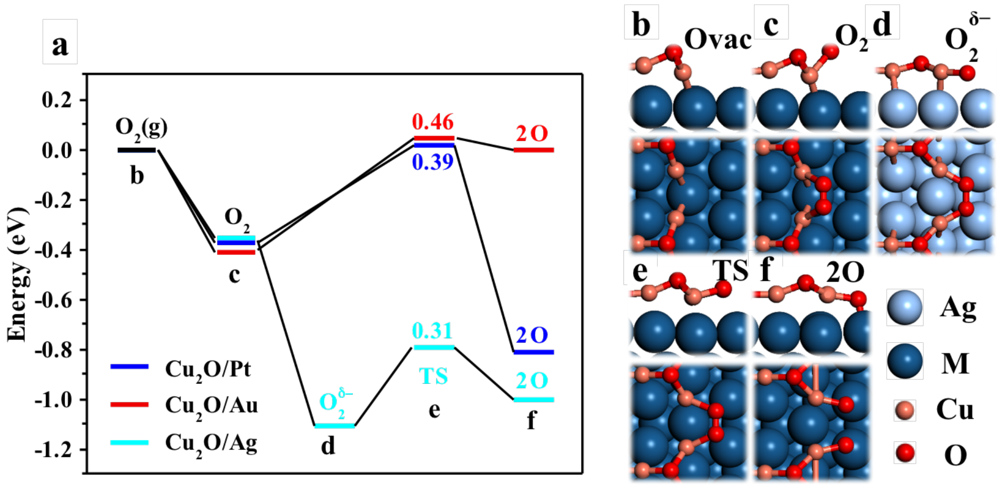


**Supplementary Figure 17.** DFT-calculated oxygen dissociation reaction on supported $Cu_2O$ NSs on M(111). (a) Potential energy diagram for the reaction of $O_2$ dissociation at exposed Cu sites (with oxygen vacancy) at the edge of supported $Cu_2O$ NSs. (b-f) The corresponding optimized structures are displayed in (b-f) and labelled in (a). In (a), the value of barriers are labelled next to their corresponding transition states (unit: eV).

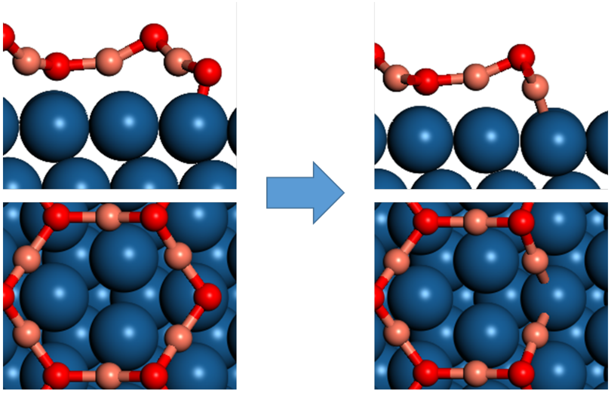

**Supplementary Figure 18.** The schematic of the formation of the Cu-Pt bond after edge O was removed. Red, coral pink and blue balls are O, Cu and Pt atoms, respectively. For $Cu_2O$/Au and $Cu_2O$/Ag, similar Cu-M bonds are observed.
